# Effectiveness of ultrasonic measurement for the hyomental distance and distance from skin to epiglottis in predicting difficult laryngoscopy in children

**DOI:** 10.1007/s00330-023-09757-z

**Published:** 2023-05-31

**Authors:** Zhenwei Zheng, Xia Wang, Ruiming Du, Qingda Wu, Lu Chen, Wuhua Ma

**Affiliations:** 1 Department of Anesthesiology, Second Affiliated Hospital of Shantou University Medical College, Shantou, China; 2 Department of Anesthesiology, First Affiliated Hospital of Guangzhou University of Chinese Medicine, Guangzhou, China

**Keywords:** Ultrasonic, Epiglottis, Airway management, Child, Laryngoscopy

## Abstract

**Objectives:**

Studies have shown that some ultrasonic indicators can predict difficult airways in adults to an extent. However, whether ultrasonic parameters can be used to predict difficult airways in children is unclear. This study investigated the predictive value of several ultrasonic indices for difficult laryngoscopy (DL) in children.

**Methods:**

Pediatric patients aged 5 to 12 years who underwent endotracheal intubation under general anesthesia were enrolled. The hyomental distance in the extended position (HMDE), tongue thickness, midsagittal tongue cross-sectional area, tongue width, and distance from skin to epiglottis (DSE) were measured by ultrasound before anesthesia. The study end point was DL. The receiver operating characteristic curve was used to evaluate the predictive value of each parameter.

**Results:**

Three hundred and ten children were included in the final analysis, and fifteen (4.8%) children had DL. The shortened HMDE assessed by ultrasound could help identify children aged 5 to 12 years with DL (5–8 years: area under the curve (AUC) 0.74, sensitivity 0.88, specificity 0.60; 9–12 years: AUC 0.72, sensitivity 0.71, specificity 0.83). An increased DSE could help identify children aged 5 to 8 years with DL (AUC 0.76, sensitivity 0.88, specificity 0.69).

**Conclusions:**

Ultrasonic measurement of the HMDE can be used to predict DL in children aged 5 to 12 years. The DSE measured by ultrasound can be used to predict DL in children aged 5 to 8 years.

**Clinical relevance statement:**

The hyomental distance and the distance from skin to epiglottis measured by ultrasound can be used to predict difficult laryngoscopy in children, which can help reduce serious complications caused by unanticipated difficult airways in children during anesthesia.

**Key Points:**

*• Ultrasonic measurement of the hyomental distance in the extended position may be an effective predictor of difficult laryngoscopy in children aged 5 to 12 years.*

*• The distance from skin to epiglottis measured by ultrasound can be used to predict difficult laryngoscopy in children aged 5 to 8 years.*

*• Preoperative airway assessment using ultrasound can be effectively applied in children and has a great application prospect.*

## Introduction

Unanticipated difficult laryngoscopy (DL) is a major cause of difficult tracheal intubation, which may lead to severe hypoxia and even death. Accurate airway assessment may reduce the incidence of difficult endotracheal intubation and related complications. However, to date, there is no authoritative parameter that can accurately predict difficult airways in children [1].

Ultrasonography has been widely used in airway management, such as the prediction of difficult airways [2], the localization of cricothyroid membranes [3, 4], the selection of tracheal tubes [5, 6], and the evaluation of patients with a full stomach [7]. Among them, ultrasonic measurements of the dimensions of certain airway parameters have predictive value for difficult airways; therefore, airway ultrasonography has been proposed as an adjunct to difficult airway prediction [2, 8].

In recent years, preoperative ultrasonography has been increasingly used to be predict certain diseases in children [9]. Many studies have shown that some ultrasonic parameters can predict difficult airways in adults [10–15], such as tongue thickness [10], hyomental distance in the extended position (HMDE) [11], distance from skin to epiglottis (DSE) [12], tongue volume [13, 14], midsagittal tongue cross-sectional area (TCSA), and tongue width [11, 15]. However, whether these parameters can be used to predict difficult airways in children is unclear. Therefore, the authors aimed to investigate which of the above ultrasonic indicators can predict DL in children.

## Materials and methods

This study was approved by the Ethics Committee of Second Affiliated Hospital of Shantou University Medical College (No. 2018–33). It was registered into chictr.org.cn on November 21, 2018 (No.ChiCTR1800019655). Written informed consent forms were signed by the parents of pediatric patients before enrollment in the study.

### Patients

Pediatric patients aged 5 to 12 years, with an American Society of Anesthesiologists grade I or II and undergoing tracheal intubation under general anesthesia, were included in this prospective observational study. Pediatric patients with the following conditions were excluded from the study: (1) failure to cooperate with clinical airway assessments or ultrasonic measurements; (2) maxillofacial trauma or tumor; (3) a large mass, tumor, or scar under the chin, or a history of neck surgery; (4) identified difficult airway or difficult airway history; or (5) cancelation of tracheal intubation for a nondifficult airway reason.

### Clinical airway assessments

Classic clinical airway assessments were performed the day before the operation, including interincisor distance (IID), thyromental distance (TMD), and modified Mallampati score (MMS) [16, 17]. The IID was the distance between the upper and lower incisors at the midline with the mouth fully opened. The TMD was the distance from the upper thyroid cartilage notch to the bony point of the mentum with the neck fully extended. The MMS was graded according to the visibility of the pharyngeal structure when the pediatric patient sat upright, opened the mouth, and protruded the tongue to the maximum, the grade ranged from 1 to 4.

### Sonography

**Fig. 1:**
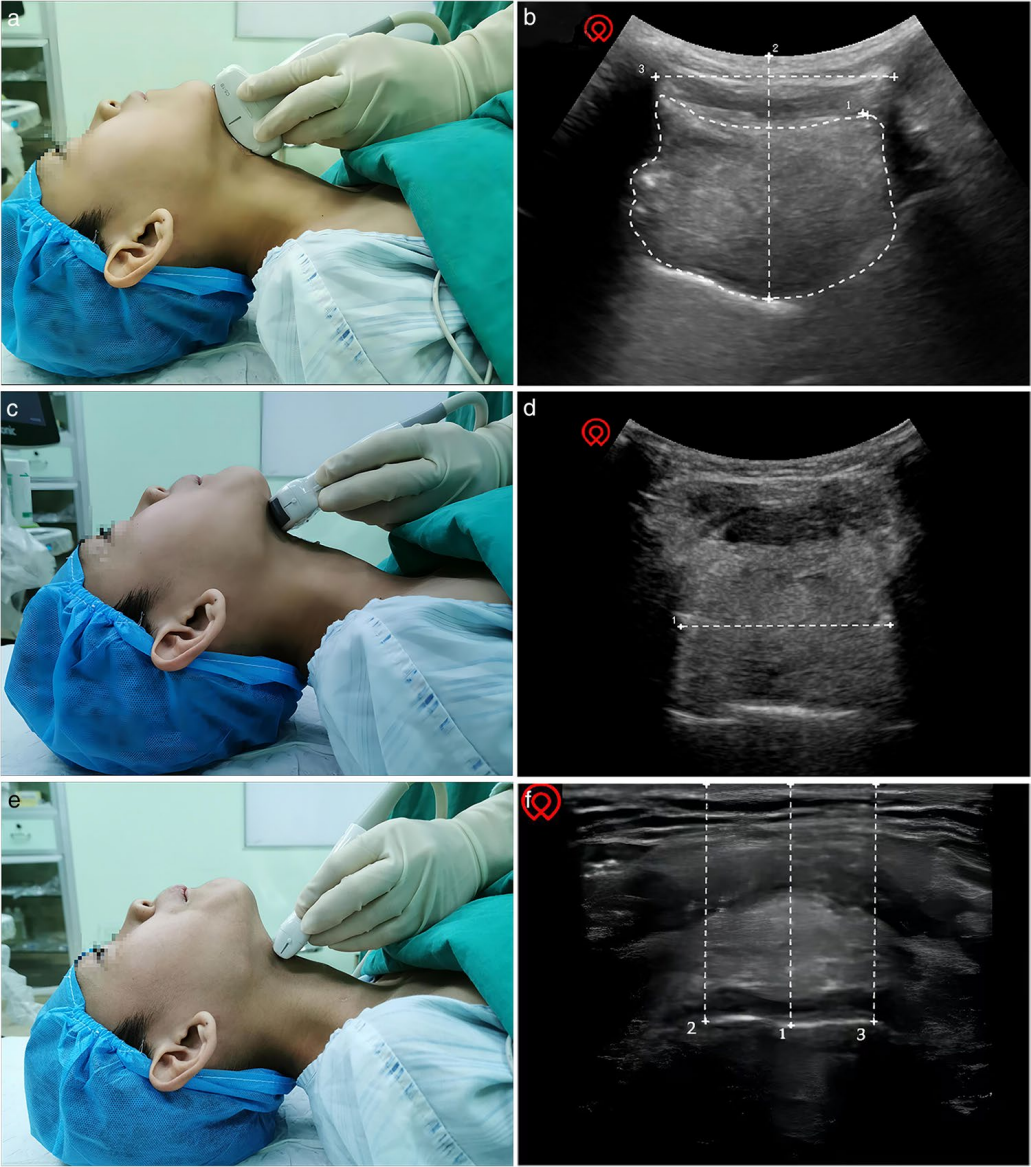
Positioning of the ultrasound probe and sonographic images of the submandibular area. **a** The low-frequency probe was placed under the chin in the midsagittal plane. **b** White dotted area: (1) tongue cross-sectional area (TCSA); (2) tongue thickness; (3) hyomental distance in the extended position (HMDE). **c** The low-frequency probe was placed transversely under the chin to obtain an entire tongue contour. **d** White dotted area: tongue width. **e** The high-frequency probe was placed transversely at the level of thyrohyoid membrane. **f** White dotted area: the vertical distances from the submental skin to the midpoint of the epiglottis and to the left and right extremities of the epiglottis, the mean of them was the distance from skin to epiglottis (DSE)

After full communication with the pediatric patients and their parents, the enrolled children underwent routine ultrasonic measurement in the operation room before anesthesia induction, while those younger or uncooperative children underwent ultrasonic measurements in a transitional waiting hall of the operating room accompanied by their parents. An ultrasound machine (Navi, Shenzhen Wisonic Medical Technology Co., Ltd.) with a low-frequency convex array probe (1–5 MHz) and a high-frequency linear array probe (6–15 MHz) was used for sonography. An anesthesiologist with experience in ultrasonography performed the sonography and ultrasonic image acquisition, and took measurements on the ultrasound images in his free time. To obtain uniform ultrasonic images, all children were placed in a supine position with their heads fully tilted back without a pillow, and asked to keep their mouths closed with the tip of their tongue lightly touching their incisors and not to make a sound. Then, the low-frequency probe was first placed under the chin in the midsagittal plane (Fig. 1a) and adjusted to show a clear view of the entire tongue outline and the border of the mandible and hyoid bone on the screen. The image was frozen and stored (Fig. 1b). On the image, the cross-sectional area of the tongue was measured by depicting the trajectory of the tongue [11, 15]. Then, the tongue thickness was measured, which was the maximum vertical distance from the submental skin to the tongue surface[10]. After that, the HMDE was measured, which was the distance from the lower border of the mentum of the mandible to the upper border of the hyoid bone (Fig. 1b) [11, 18]. Then, the probe was rotated 90 degrees and placed transversely under the chin and the midpoint of the probe was on the midline (Fig. 1c). After that, the probe was adjusted to obtain as complete a tongue contour as possible, and the ultrasonic image was also frozen and stored. The distance between the most distant points on the middle surface of the tongue was measured on the image and defined as tongue width (Fig. 1d) [11, 15]. Finally, the high-frequency probe was placed transversely at the level of the thyrohyoid membrane and adjusted to obtain a clear view of the epiglottis (Fig. 1e). The epiglottis was visible as a curvilinear hypoechoic structure, and the image was frozen and stored. Then, the vertical distances from the submental skin to the midpoint of the epiglottis and to the left and right extremities of the epiglottis were measured, and the DSE was the mean of the above three vertical distances (Fig. 1f) [12]. The anesthesiologist who performed the sonography was blinded to the results of the Cormack-Lehane laryngoscopic grading.

### Induction of general anesthesia

After all ultrasonic measurements were completed, peripheral venous access was routinely established for the pediatric patients. Children who were afraid of venipuncture inhaled 8% sevoflurane until they fell asleep, and then peripheral venous access was established. Non-invasive blood pressure, oxygen saturations, electrocardiogram, respiration, and end-tidal carbon dioxide partial pressure were regularly monitored. Preoxygenation was administered for at least 2 min. Then, midazolam (0.1 mg·kg^−1^), propofol (2 mg·kg^−1^), fentanyl (0.004 mg·kg^−1^), and cisatracurium 0.2 (mg·kg^−1^) were administered intravenously for anesthesia induction. Mask ventilation was administered for 3 min after spontaneous breathing was weakened. Finally, one of four designated anesthesiologists with more than 5 years of experience performed the laryngoscopies using an appropriately sized (size 2 to 4) curved Macintosh blade, allowing the application of external laryngeal pressure to ameliorate glottic exposure. Then, an appropriately sized endotracheal tube was inserted into the trachea. The anesthesiologist who performed the laryngoscopy was blinded to the results of the ultrasonic measurements.

### Study end points

The Cormack-Lehane grading [19] was used to score the visibility of the glottis during each laryngoscopy. Grade 3 or 4 was considered to be a difficult laryngoscopy. According to the age and the results of the Cormack-Lehane grading, the enrolled children were divided into a 5- to 8-year-old DL group (DL5 group) and a 5- to 8-year-old nondifficult laryngoscopy group (NDL5 group), a 9- to 12-year-old DL group (DL9 group), and a 9- to 12-year-old nondifficult laryngoscopy group (NDL9 group). Alternative techniques and tools were permitted when laryngoscopy was difficult. If an emergency airway was encountered, the anesthesiologist asked for help immediately and ventilated the children first using a noninvasive airway tool or method such as a laryngeal mask. If ventilation is not possible, a surgical airway should be established as soon as possible [20].

### Statistical analysis

Statistical analysis was performed by SPSS software version 19.0. Continuous variables with a normal distribution are presented as the means ± standard deviations, and differences in the means between the two groups were compared by Student’s *t* test. Categorical variables are expressed as numbers, and differences in the means between the two groups were compared by the chi-square test or Fisher’s exact test. All comparisons were two-sided tests, and a *p* < 0.05 was considered to be significant.

Receiver operating characteristic (ROC) curve analysis and Youden’s index were used to determine the criteria for predictive variables to predict DL. The area under the curve (AUC) was used to assess the diagnostic validity of variables to predict DL. Then, the accuracy, sensitivity, specificity, positive predictive value (PPV), and negative predictive value (NPV) of the predictive variables were calculated by QuickCalcs software.

There have been few studies on sonographic measurements of airway indices in children, and the available literature is lacking. Therefore, a preliminary experiment was designed, and the data from the preliminary experiment showed that the incidence of difficult laryngoscopy in children in our institution was approximately 5%. Then, the sample size was estimated using PASS software version 11.0 based on data from the preliminary experiment [21, 22]. Considering a type I error (alpha) of 0.05, a power (1-beta) of 0.8 [22]. A sample size of at least 160 was required to detect differences in these ultrasonic parameters between the pediatric patients aged 5 to 8 years with and without difficult laryngoscopy, while a sample size of at least 140 was needed to detect differences in these ultrasonic parameters between the pediatric patients aged 9 to 12 years with and without difficult laryngoscopy.

## Result

**Fig. 2:**
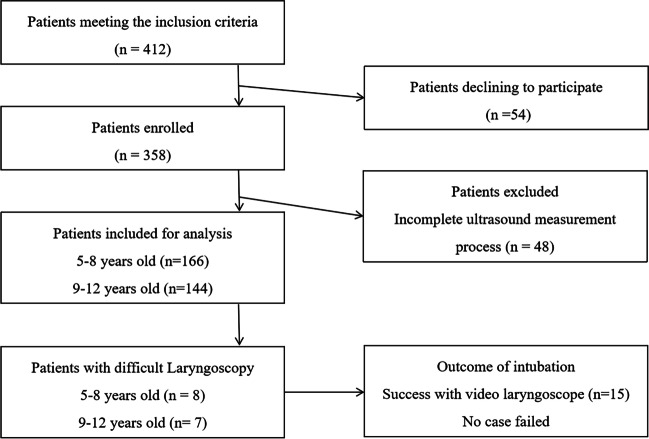
Study flow chart and patient outcomes

There were 358 pediatric patients enrolled in this study during the observation period. Forty-eight children were excluded, and 310 children were included in the final analysis, including 166 children aged 5 to 8 years and 144 children aged 9 to 12 years. Figure 2 shows the study flow chart and outcomes of the pediatric patients. A total of 15 (4.8%) children experienced DL: 8 (4.8%) in the DL5 group and 7 (4.9%) in the DL9 group. All 15 patients were successfully intubated via a video laryngoscope, and no patients experienced difficult mask ventilation.

**Fig. 3:**
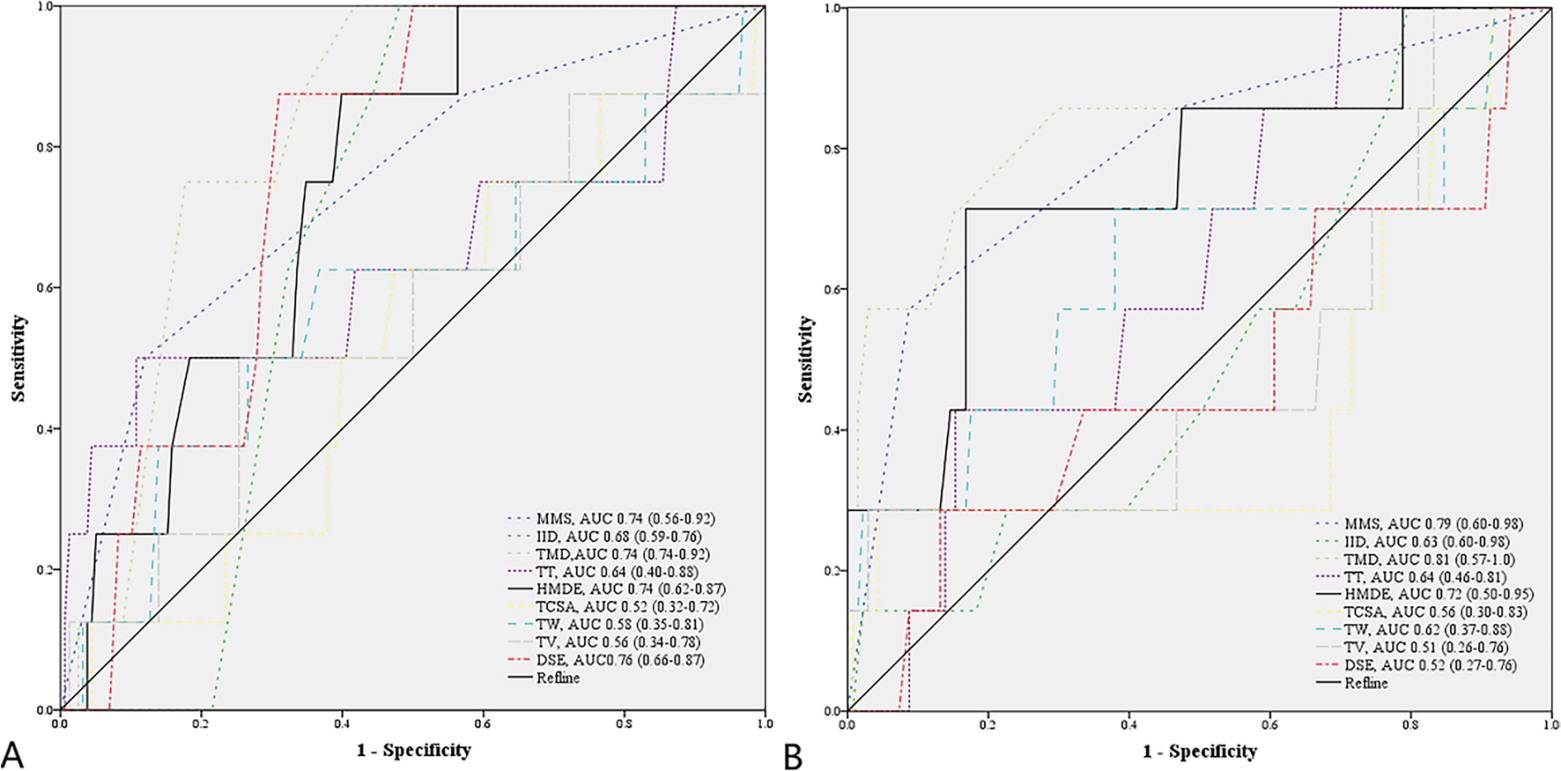
**A** Receiver operating characteristic curve analyses of airway assessment indicators and their areas under the curve (AUCs, values and 95% confidence intervals) for predicting difficult laryngoscopy in children aged 5 to 8 years (**A**) and in children aged 9 to 12 years (**B**)

No significant differences were observed in the demographic variables between the DL5 group and NDL5 group or between the DL9 group and NDL9 group (Table 1). In the univariate analysis, there were significant differences in MMS, TMD, and one ultrasonic indicator (HMDE) for predicting DL between the DL5 group and NDL5 group and between the DL9 group and NDL9 group. Another ultrasonic indicator (DSE) for predicting DL was significantly different between the DL5 group and NDL5 group. All of these indicators were regarded as effective predictors of DL in children aged 5 to 8 years or children aged 9 to 12 years. The ROC curve analysis showed that all the AUCs of MMS, TMD, HMDE, and DSE in children aged 5 to 8 years and MMS, TMD, and HMDE in children aged 9 to 12 years were between 0.7 and 0.9 (Table 1). Figure 3 shows the ROC curves of the above effective indicators in children aged 5 to 8 years (A) and in children aged 9 to 12 years (B). Tables 2 and 3 show the optimal cutoffs for effective predictors of DL in children aged 5 to 8 years and in children aged 9 to 12 years, which were determined by maximizing Youden’s index: sensitivity + specificity − 1. Moreover, Tables 2 and 3 mainly show the accuracy, sensitivity, specificity, PPV, NPV, and their 95% confidence intervals for DL when the effective predictors were greater or less than the optimal cutoff value.

**Table 1:** Comparisons of pediatric patients with and without difficult laryngoscopy

Variable	DL5 (*n* = 8)	NDL5 (*n* = 158)	*p* value	AUC (95% CI)	DL9 (*n* = 7)	NDL9 (*n* = 137)	*p* value	AUC (95% CI)
Sex (male/female, *n*)	3/5	112/46	0.109 (C)	–	5/2	91/46	1.0 (C)	–
Age (years)	6.1 ± 1.1	6.3 ± 1.1	0.612 (T)	0.55 (0.35–0.75)	10.7 ± 1.1	10.5 ± 1.1	0.576 (T)	0.56 (0.36–0.76)
BMI (kg/m^2^)	16.2 ± 3.1	15.1 ± 2.1	0.182 (T)	0.60 (0.37–0.83)	18.4 ± 3.8	16.9 ± 3.2	0.247 (T)	0.62 (0.39–0.85)
MMS (1/2/3/4)	1/3/4/0	68/71/19/0	0.012 (C)	0.74 (0.56–0.92)	1/2/4/0	73/52/12/0	0.001 (C)	0.79 (0.60–0.98)
IID (cm)	3.5 ± 0.1	3.8 ± 0.5	0.119 (T)	0.68 (0.59–0.76)	4.1 ± 0.6	4.2 ± 0.6	0.573 (T)	0.63 (0.60–0.98)
TMD (cm)	5.0 ± 0.3	5.7 ± 0.7	0.006 (T)	0.83 (0.74–0.92)	5.5 ± 1.0	6.4 ± 0.6	< 0.001 (T)	0.81 (0.57–1.0)
Tongue thickness (cm)	4.7 ± 0.5	4.4 ± 0.4	0.065 (T)	0.64 (0.40–0.88)	5.1 ± 0.4	4.9 ± 0.5	0.251 (T)	0.64 (0.46–0.81)
HMDE (cm)	3.7 ± 0.2	4.0 ± 0.4	0.030 (T)	0.74 (0.62–0.87)	4.1 ± 0.6	4.6 ± 0.5	0.012 (T)	0.72 (0.50–0.95)
TCSA (cm^2^)	12.2 ± 1.8	12.1 ± 1.7	0.998 (T)	0.52 (0.32–0.72)	15.6 ± 3.1	15.4 ± 2.1	0.848 (T)	0.56 (0.30–0.83)
Tongue width (cm)	3.7 ± 0.4	3.6 ± 0.3	0.499 (T)	0.58 (0.35–0.81)	4.2 ± 0.6	4.0 ± 0.4	0.071 (T)	0.62 (0.37–0.88)
Tongue volume (cm^3^)	45.8 ± 11.2	44.3 ± 8.3	0.644 (T)	0.56 (0.34–0.78)	67.1 ± 23.0	61.5 ± 12.3	0.285 (T)	0.51 (0.26–0.76)
DSE (cm)	1.6 ± 0.1	1.5 ± 0.1	0.020 (T)	0.76 (0.66–0.87)	1.6 ± 0.2	1.7 ± 0.2	0.783 (T)	0.52 (0.27–0.76)

*Abbreviation*: *(T)* two sided *t*-test, *(C)* chi-square test, *BMI* body mass index, *MMS* modified Mallampati score, *IID* interincisor distance, *TMD* thyromental distance, *HMDE* hyomental distance in the extended position, *TCSA* tongue cross-sectional area, *DSE* distance from skin to epiglottis, *AUC* area under the curve, *CI* confidence interval

**Table 2:** Diagnostic validity profiles of effective predictors of difficult laryngoscopy in children aged 5–8 years

Predictors	Accuracy (95% CI)	Sensitivity (95% CI)	Specificity (95% CI)	PPV (95% CI)	NPV (95% CI)
MMS > 2	0.86 (0.80, 0.91)	0.50 (0.21, 0.78)	0.88 (0.82, 0.92)	0.17 (0.06, 0.38)	0.97 (0.93, 0.99)
TMD < 5.6 cm	0.60 (0.53, 0.67)	1.0 (0.63, 1.0)	0.58 (0.50, 0.66)	0.11 (0.05, 0.20)	1.0 (0.95, 1.0)
HMDE < 3.9 cm	0.61 (0.54, 0.68)	0.88 (0.51, 1.0)	0.60 (0.52, 0.67)	0.10 (0.05, 0.20)	0.99 (0.94, 1.0)
DSE > 1.5 cm	0.70 (0.62, 0.76)	0.88 (0.51, 1.0)	0.69 (0.61, 0.76)	0.12 (0.06, 0.24)	0.99 (0.94, 1.0)

*Abbreviation*: *MMS* modified Mallampati score, *TMD* thyromental distance, *HMDE* hyomental distance in the extended position, *DSE* distance from skin to epiglottis, *CI* confidence interval, *PPV* positive predictive value, *NPV* negative predictive value

**Table 3:** Diagnostic validity profiles of effective predictors of difficult laryngoscopy in children aged 9–12 years

Predictors	Accuracy (95% CI)	Sensitivity (95% CI)	Specificity (95% CI)	PPV (95% CI)	NPV (95% CI)
MMS > 2	0.90 (0.68, 0.94)	0.57 (0.25, 0.84)	0.91 (0.85, 0.95)	0.25 (0.10, 0.50)	0.98 (0.93, 1.0)
TMD ≤ 5.8 cm	0.84 (0.77, 0.89)	0.71 (0.35, 0.92)	0.85 (0.78, 0.90)	0.19 (0.08, 0.38)	0.98 (0.94, 1.0)
HMDE < 4.2 cm	0.83 (0.76, 0.88)	0.71 (0.35, 0.92)	0.83 (0.76, 0.89)	0.18 (0.07, 0.36)	0.98 (0.94, 1.0)

*Abbreviation*: *MMS* modified Mallampati score, *TMD* thyromental distance, *HMDE* hyomental distance in the extended position, *CI* confidence interval, *PPV* positive predictive value, *NPV* negative predictive value

## Discussion

This study shows that a short HMDE measured by ultrasound may help identify children aged 5 to 12 years with DL, and a long DSE assessed by ultrasound may help identify children aged 5 to 8 years with DL. As we know, the ROC curve is a graph closely related to sensitivity and specificity, and the AUC of the ROC curve can effectively evaluate the diagnostic validity of parameters. In this study, all the AUC values of the MMS, TMD, HMDE, and DSE in children aged 5 to 8 years and the MMS, TMD, and HMDE in children aged 9 to 12 years were between 0.7 and 0.9. The above results may indicate that both a high MMS and a short TMD have medium predictive value for DL in children aged 5 to 12 years; as can be seen from Tables 2 and 3, MMS has high accuracy and specificity in predicting DL in children aged 5 to 12 years, but low sensitivity, while TMD has high sensitivity and low specificity in predicting DL in children aged 5 to 8 years, and high specificity and moderate sensitivity in predicting DL in children aged 9 to 12. Therefore, MMS and TMD remain useful predictors of DL for children aged 5 to 12 years, the combined use of MMS and TMD will better predict DL in children aged 5 to 12 years, and preoperative clinical airway assessment may also be important for anesthesiologists to identify children with DL. Furthermore, the above results may show that a short HMDE has medium predictive value for DL in children aged 5 to 12 years, and a long DSE has medium predictive value for DL in children aged 5 to 8 years.

In recent years, airway assessment by ultrasound has been proposed as a valuable assessment method and an adjunct to clinical airway assessment [2, 8]. In several studies, researchers have demonstrated that HMDE was associated with difficult airways in adults [11, 18, 23], which was similar to the findings of these studies; the present study showed that a short HMDE also had medium predictive value for difficult laryngoscopy in children aged 5 to 12 years, and HMDE < 3.9 cm could be used to predict difficult laryngoscopy in children aged 5 to 8 years with a sensitivity of 0.88 and a specificity of 0.60 (Table 2), while HMDE < 4.2 cm could be used to predict difficult laryngoscopy in children aged 9 to 12 years with a sensitivity of 0.71 and a specificity of 0.83 (Table 3). From Tables 2 and 3, it can be seen that HMDE and TMD have similar diagnostic values in predicting DL in children aged 5 to 12 years, indicating that HMDE and TMD have a high correlation. On the other hand, studies have shown that a long DSE could be used to predict difficult laryngoscopy in adults [15, 23]; this study showed that a long DSE assessed by ultrasound could be used to predict difficult laryngoscopy in children aged 5 to 8 years, and a DSE > 1.5 cm could help identify children aged 5 to 8 years with difficult laryngoscopy with a sensitivity of 0.88 and a specificity of 0.69. However, the results of this study indicated that a long DSE was not associated with difficult laryngoscopy in children 9 to 12 years. Therefore, further research may be needed to confirm the predictive value of DSE in predicting DL in children. Yao and colleagues found that tongue thickness measured by ultrasound was an independent predictor of difficult laryngoscopy in adults [10]; furthermore, several studies have shown that the ultrasonic measurement of some tongue indicators (tongue volume, TCSA, and tongue width), as well as advanced age and male sex, was all associated with difficult laryngoscopy in adults [12–15]. However, the present study showed that none of these indicators were associated with difficult laryngoscopy in children aged 5 to 12 years. The above differences may be due to the different anatomical conditions in children and adults.

This study has some limitations. First, this is a single-center study, and one single ethnic group (Han Chinese) was included, so there may be some limitations on the representativeness of the results. Second, ultrasonic measurement requires the child to keep the head back as much as possible and the mouth closed while awake, and the ultrasound probe should be placed above the thyrohyoid membrane to measure the distance from the skin to the epiglottis, which can easily cause discomfort in children. Most children younger than 5 years of age were often unable to cooperate and were therefore excluded from the study, so only children aged 5 to 12 years were included in this study. Third, the limitation of grouping. The incidence of difficult laryngoscopy in children was very low; if the pediatric patients were grouped every 2 years, a large sample size would be required to detect significant differences in certain indicators between difficult and nondifficult laryngoscopy cases in each age group. However, the collection of pediatric case data was very difficult. If the pediatric patients were grouped every 3 years, the grouping would be uneven. Therefore, the pediatric patients were eventually divided into two groups every 4 years, the 5–8-year-old group and the 9–12-year-old group. Fourth, Tables 2 and 3 show that all effective predictors of DL in children had very low PPVs and very high NPVs, which might be due to the low incidence of DL in children. Furthermore, the PPV and NPV were calculated with the sampling prevalence of this study, so they may not be generalizable.

## Conclusions

This study showed that the HMDE measured by ultrasound may be an effective predictor of DL in children aged 5 to 12 years. The measurement of HMDE does not require that children actively participate in the process which thus may have great application prospects in predicting difficult airways in children. Ultrasonic measurement of DSE may be a valuable predictor of difficult laryngoscopy in children aged 5 to 8 years, but further research may be needed to determine its predictive value for children with difficult airways. Furthermore, both MMS and TMD are useful predictors of DL for children aged 5 to 12 years, and combining HDME or TMD with MMS for preoperative airway assessment may improve the predictive ability of DL in children aged 5 to 12 years, while DSE in combined with TMD and MMS may also better predict DL in children aged 5 to 8 years.

## Abbreviations

AUC: Area under the curve
DL: Difficult laryngoscopy
DSE: Distance from skin to epiglottis
HMDE: Hyomental distance in the extended position
IID: Interincisor distance
MMS: Modified Mallampati score
NPV: Negative predictive value
PPV: Positive predictive value
ROC: Receiver operating characteristic
TCSA: Tongue cross-sectional area
TMD: Thyromental distance

## Declarations

### Guarantor

The scientific guarantor of this publication is Professor Wuhua MA from the Department of Anesthesiology, the First Affiliated Hospital of Guangzhou University of Chinese Medicine.

### Statistics and biometry

Dr. Dawei YANG (School of Public Health, Sun Yat-sen University) kindly provided statistical advice for this manuscript.

### Methodology

- prospective
- observational study
- performed at one institution
